# Prediction of dumping after oesophageal cancer surgery

**DOI:** 10.2340/1651-226X.2026.45044

**Published:** 2026-03-05

**Authors:** Pernilla Lagergren, Laima Kampa, Kalle Mälberg, William Jebril, Johan Hardvik Åkerström, Asif Johar, Anna Schandl

**Affiliations:** a Surgical Care Science, Department of Molecular Medicine and Surgery, Karolinska Institutet, Karolinska University Hospital, Stockholm, Sweden; b Department of Surgery and Cancer, Imperial College London, London, United Kingdom; c Laboratory of Sports and Nutrition Research, Riga Stradiņš University, Riga, Latvia; d Department of Human Physiology and Biochemistry, Riga Stradiņš University, Riga, Latvia; e Upper Gastrointestinal Surgery, Department of Molecular medicine and Surgery, Karolinska Institutet, and Karolinska University Hospital, Stockholm, Sweden; f Department of Perioperative- and Intensive Care, Södersjukhuset, Sweden; g Department of Clinical Sciences and Education, Karolinska Institutet, Södersjukhuset, Sweden

**Keywords:** oesophageal neoplasms, dumping syndrome, prediction models, postoperative complications, survey, questionnaires

## Abstract

**Introduction:**

Dumping syndrome is a common consequence following oesophagectomy and may lead to reduced food intake, resulting in subsequent weight loss, and negatively impact health-related quality of life. This study aimed to develop a predictive model to facilitate preventive treatment planning by identifying individuals at an increased risk of developing dumping syndrome.

**Materials and methods:**

Data were obtained from a nationwide, population-based cohort of patients who underwent oesophagectomy for cancer between 2013 and 2020. Patient and clinical characteristics were retrieved from national health registries and medical records. Dumping symptoms were self-reported 1 year after surgery. Multivariable regression models provided odds ratios (OR) with 95% confidence intervals (CI). Model performance was evaluated using the area under the receiver operating characteristic curve (AUC).

**Results:**

Among the 384 individuals, 41 (11%) developed significant symptoms of dumping. The following variables that increased the probability of developing dumping were included in the final prediction model: younger age, female sex, higher preoperative body mass index, no neoadjuvant therapy, Charlson Comorbidity Index score > 0, open oesophagectomy, cervical anastomosis, and less severe postoperative complications (lower Clavien Dindo grade). For the prediction of early dumping symptoms, the AUC for the total cohort was 0.74 (95% CI: 0.64–0.83) and after cross-validation, 0.62 (95% CI: 0.53–0.72). For early and late symptoms, the AUC was 0.75 (95% CI: 0.67–0.83), and for the cross-validated model, 0.65 (95% CI: 0.56–0.74).

**Interpretation:**

This study offers insights into factors associated with dumping syndrome after oesophagectomy. The prediction model showed modest ability to distinguish between high and low-risk patients, and should therefore be interpreted as supportive rather than definitive. Its potential value lies in complementing clinical judgement, informing structured follow-up and guiding future research.

## Introduction

Dumping syndrome is a complication that can occur after oesophagectomy and is characterised by unpleasant symptoms experienced following ingestion of a meal. Dumping syndrome is considered to be a consequence of the rapid transit of gastric chyme into the small intestine [1, 2], attributed to anatomical changes of the gastrointestinal tract and disruption of its neural innervation following oesophagectomy [3]. Symptoms are believed to be related to hyperosmolar gastric chyme, causing rapid fluid shifts into the intestinal lumen and increased secretion of several gastrointestinal hormones, including incretins such as glucagon-like peptide-1, enhancing insulin secretion and potentially causing reactive hypoglycaemia [4].

Early dumping symptoms commonly include abdominal cramps, diarrhoea, palpitations, and nausea and occur within 1 hour after a meal. Symptoms of early dumping may occur in isolation or together with late dumping symptoms. Late dumping symptoms typically occur 1–3 hours after a meal and are often associated with hypoglycaemia. Symptoms of late dumping commonly involve dizziness, faintness, cold sweats and hunger [5–7]. A recent systematic review and meta-analysis demonstrated a pooled incidence estimate of dumping syndrome after oesophagectomy of 27% worldwide and 39% in Western countries [8]. However, this analysis did not distinguish between early and late dumping symptoms.

Dumping symptoms may lead to reduced food intake, resulting in subsequent weight loss, and negatively impact the survivors’ health-related quality of life [6, 9, 10]. Certain surgical factors are believed to increase the risk of post-oesophagectomy dumping, including pyloric drainage procedures [11, 12] and vagotomy [13, 14]. More severe dumping syndrome has been associated with cervical anastomosis compared to intrathoracic anastomosis, as per a few studies, but others have shown no differences between the two [7]. Moreover, no correlation has been found between conduit type, conduit position, width of the conduit, or surgical technique (open or minimally invasive) [7]. Although certain surgical approaches have been associated with fewer dumping symptoms, they have been reported in only a small number of studies with methodological heterogeneity and often contradictory findings [7]. This study aimed to develop a predictive model to facilitate preventive treatment planning by identifying individuals at increased risk of developing dumping syndrome.

## Materials and methods

### Study design

This study was based on data from a population-based cohort study entitled: Oesophageal Surgery on Cancer patients – Adaptation and Recovery (OSCAR), which is described in detail elsewhere [15]. In brief, patients were identified through collaboration with all eight pathology departments in the hospitals where these procedures are conducted in Sweden. The entire cohort consists of 1013 patients who underwent oesophageal cancer surgery between 01 January 2013 and 30 June 2020 were alive 1 year after surgery and had a formal home address. All included patients were followed up by a research nurse who visited them in their homes 1 year after surgery and guided them through self-reported computer-based questionnaires. Clinical data were extracted from medical records in accordance with a predefined study protocol to ensure consistency and standardisation. The collected data included tumour histology, site and stage, cancer treatment, and postoperative complications. An independent person performed cross-validation of randomly selected protocols. Data on patient characteristics were collected by linking the unique person identification number assigned to each Swedish resident to national health data registries. Socio-demographic information was obtained via linkage to the Longitudinal Integration Database for Health Insurance and Labour Market, which has been in operation since 1990 and is updated annually [16]. For information on comorbidities, the patients were linked to the Swedish Patient Registry [17] and the Swedish Cancer Registry [18]. Comorbidities were classified using the well-validated Charlson Comorbidity Index score [19, 20]. The Swedish Register of the Total Population [21], which holds nearly 100% complete nationwide information, was used to retrieve survival data. The study was approved by the Regional Ethical Review Board in Stockholm, Sweden (2013/844-31/1), and all participants provided written informed consent. Reporting followed the TRIPOD+AI statement guide [22].

### Outcomes

The outcome was the presence of early, significant dumping symptoms with or without the co-occurrence of late significant symptoms. Information on the presence and intensity of dumping symptoms was obtained 1 year after surgery using a study-specific questionnaire (OSCAR dumping questionnaire). Patients were asked to report symptom intensity on an average day. The OSCAR dumping questionnaire is based on the Arts dumping questionnaire [6], the most commonly used questionnaire for dumping. There are 10 common symptoms of dumping syndrome, with response alternatives yes/no, with a 4-point intensity scale: (1) not at all, (2) a little, (3) quite a bit, or (4) very much. Furthermore, patients reported whether they had experienced the symptoms prior to surgery. Symptoms of dumping that were also present before surgery, but not related to eating a meal, were not regarded as dumping symptoms. Additionally, two open-ended questions assessed the occurrence of symptoms throughout the day and their relation to meals. Early or late dumping was inferred from the responses to the two open-ended questions. The responses were verified by the research nurse who guided the questionnaire-based assessment. Early dumping was defined as symptoms within 60 minutes after a meal, and late dumping as symptoms occurring 1–3 hours after a meal. The symptoms were further divided according to intensity, with significant symptoms presented as having at least two symptoms rated as ‘quite a bit’ or ‘very much’, and less symptomatic cases serving as the reference group.

### Candidate predictors

The selection of candidate predictors was based on literature and clinicians’ input. These variables were age at surgery (continuous) [7, 9, 23], biological sex (men/women) [23], preoperative body mass index (BMI) [7], tobacco smoking (ever/never), alcohol consumption (ever/never), comorbidity (assessed with Charlson Comorbidity Index score 0, 1, > 2) [24], surgery technique-open oesophagectomy (transthoracic or transhiatal), minimally invasive oesophagectomy (MIO) (thoracoscopic-laparoscopic), or hybrid minimally invasive oesophagectomy (HMIO) (thoracoscopic/open abdomen or laparoscopic/open chest) [7, 24]; type of substitute (gastric tube/colon/ileocolic segment), location of anastomosis (cervical/intrathoracic) [7], gastrectomy (total/partial), and histology type (adenocarcinoma or Barrett’s oesophagus with high grade dysplasia/squamous cell carcinoma) [24], neoadjuvant therapy (neoadjuvant chemotherapy, neoadjuvant chemoradiotherapy or surgery alone) [24], pyloric procedure (pyloroplasty and/or pylorotomy/postoperative pyloric dilatation) [7], pathological tumour stage (I/II/III–IV), postoperative complications (low grade – Clavien Dindo Classification (0–1, 2–3a) / high grade – Clavien Dindo Classification 3b–4).

### Statistical analysis

We analysed two separate prediction models, a first model for early significant dumping (less symptoms/significant symptoms) and a second model for early and late significant dumping symptoms (less symptoms/significant symptoms). The prediction model was built in a two-step approach. The initial model was selected by assessing the association between the primary outcome and candidate predictors using univariable logistic regression modelling by including one covariate at a time. Candidate predictors with a *p*-value < 0.1 in the univariable analysis were included in the initial multivariable prediction model. A receiver operating characteristic curve (ROC) showed the diagnostic ability of a prediction model as its discrimination cut-off varied. The ROC curve was created by plotting true positive rates against false positive rates. The area under the receiver operating characteristic curve (AUC) is a measure (between 0 and 1) of the overall accuracy of the prediction model: the higher the AUC, the better the model. In the second step, each candidate predictor from the initial step was temporarily removed one at a time while all remaining predictors were retained, and the corresponding model AUC was recalculated. Predictors that failed univariable screening were also reintroduced for evaluation. Predictors whose individual removal resulted in an AUC decrease of more than 1% were retained in the final model. To further evaluate the predictive accuracy of the model, the mean AUC of 1000 bootstrap samples were calculated. Results from the final model were presented as odds ratios (ORs) with 95% confidence intervals (CIs) and AUCs. Hosmer and Lemeshow’s Goodness-of-Fit test was calculated for the final prediction model. Missing data were handled using multiple imputation by chained equations, generating 10 imputed datasets under a missing-at-random assumption. Estimates were pooled using Rubin’s rules [25]. To identify non-linear relationships, subgroups, or candidate predictor interactions that might enhance the model, we employed classification and regression tree (CART) analysis [26]. CART recursively partitions the data into homogeneous subgroups using binary splits based on candidate predictor variables, generating an interpretable decision tree. Classification accuracy and mean standard error were presented for the training and validation models, respectively. A senior biostatistician (AJ) conducted all statistical analyses and data management using the statistical software SAS Statistical Package (version 9.4, SAS Institute Inc., Gary, NC).

## Results

In total, 1013 patients underwent oesophageal cancer resection, of which 242 died within the first year, and 154 could not be reached, leaving 617 eligible for the study. Among these, 143 patients (23%) declined consent, 55 (9%) were too sick or experienced tumour recurrence, and 4 patients had missing clinical information, leaving 415 patients in the cohort. Of these, 385 patients answered the dumping questionnaire (1 patient was removed due to an unknown tumour stage), and eventually, 384 patients were included in the study. The characteristics of the study cohort are described in Table 1. The mean age at surgery was 67 ± 8 years, and 82% were men. The majority had undergone minimally invasive or hybrid surgery. Twenty-five patients underwent a gastrectomy in addition to the oesophagectomy. In total, 41 patients (11%) had symptoms of dumping, out of which 37 reported early dumping symptoms.

**Table 1:** Characteristics of non-symptomatic versus symptomatic patients 1 year after oesophageal cancer resection.

Variables	All patients Number (%)	Less symptoms Number (%)	Significant symptoms Number (%)	Missing Number
**Total number**	384 (100)	343 (89)	41 (11)	
**Age mean ± SD**	67 ± 8	68 ± 7	64 ± 12	
**Biological sex**				
Men	314 (82)	284 (83)	30 (73)	
Women	70 (18)	59 (17)	11 (27)	
**Preop BMI, mean ±SD**	27 ± 4	27 ± 4	28 ± 5	5
**Charlson Comorbidity Index score**				
0	164 (43)	150 (44)	14 (34)	
1	132 (34)	114 (33)	18 (44)	
≥ 2	88 (23)	79 (23)	9 (22)	
**Histology**				
Adenocarcinoma or Barrett’s oesophagus with high grade dysplasia	331 (86)	297 (87)	34 (83)	
Squamous cell carcinoma	53 (14)	46 (13)	7 (17)	
**Tumour stage**				
0–I	133 (35)	120 (35)	13 (32)	
II	119 (31)	106 (31)	13 (32)	
III–IV	132 (34)	117 (34)	15 (37)	
**Surgical approach**				
Minimally invasive or hybrid	278 (72)	255 (74)	23 (56)	
Open oesophagectomy	106 (28)	88 (26)	18 (44)	
**Anastomosis location**				2
Cervical	61 (16)	52 (16)	9 (22)	
Thoracic	321 (84)	289 (84)	32 (78)	
**Substitute**				2
Colon or ileocolic segment	20 (5)	17 (5)	3 (7)	
Gastric tube	362 (95)	324 (94)	38 (93)	
**Postoperative pyloric dilatation**				37
Yes	58 (17)	53 (91)	5 (9)	
No	289 (83)	259 (90)	30 (10)	
**Neoadjuvant therapy**				
Yes	309 (80)	282 (82)	27 (66)	
No	75 (20)	61 (18)	14 (34)	
**Postoperative complications (Clavien Dindo)**				
CD 0–I	141 (37)	123 (36)	18 (44)	
CD II–IIIa	145 (38)	131 (38)	14 (34)	
CD IIIb–IV	98 (26)	89 (26)	9 (22)	

CD: Clavien Dindo Classification; SD: Standard Deviations.

### Development of the prediction model

After the univariable models, eight of the original 12 candidate predictors (age, biological sex, preoperative BMI, neoadjuvant therapy, comorbidity, surgical approach, anastomosis location, and postoperative complications) were selected for the AUC analysis (Supplementary Table 1). In the final analysis, all the selected candidate predictors were kept in the model and were removed one at a time to determine their contribution to the overall AUC. Removal of individual predictors from the full model led to modest but meaningful reductions in AUC (approximately 1–7%). The largest decreases were observed for neoadjuvant therapy (7% for early dumping and 6% for early and late dumping) and for surgical approach (2 and 5%), supporting their contribution to overall model discrimination. The final prediction model included: younger age, female sex, higher preoperative BMI, no neoadjuvant therapy, Charlson Comorbidity Index score > 0, no MIO or HMIO, cervical anastomosis, and less severe postoperative complications (lower Clavien Dindo grades), all of which increased the probability of developing dumping. ORs with 95% CIs for each model are presented in Tables 2 and 3.

**Table 2:** Candidate predictors and their association with early dumping symptoms 1 year after oesophageal cancer surgery presented as odds ratios (OR) with 95% confidence intervals (CI).

Candidate predictors	Description	Early and late dumping
		OR (95% CI)
Age at surgery	Continuous	0.96 (0.92–1.01)
Biological sex	Male	Reference
	Female	1.27 (0.54–2.99)
Preoperative BMI	Continuous	1.07 (0.99–1.16)
Neoadjuvant therapy	Yes	Reference
	No	3.27 (1.48–7.25)
Charlson Comorbidity Index score	0	Reference
	1	2.33 (0.99–5.49)
Charlson Comorbidity Index score	0	Reference
	≥ 2	1.55 (0.55–4.34)
Surgical approach	Open oesophagectomy	Reference
	Minimally invasive/hybrid	0.44 (0.21–0.94)
Anastomosis location	Thoracic	Reference
	Cervical	2.05 (0.79–5.31)
Postoperative complications	CD IIIb–IV	Reference
	CD 0–I	2.88 (1.04–8.01)
Postoperative complications	CD IIIb–IV	Reference
	CD II–IIIa	1.74 (0.62–4.84)

CD: Clavien Dindo.

**Table 3:** Candidate predictors and their association with early and late dumping symptoms 1 year after oesophageal cancer surgery presented as odds ratios (OR) with 95% confidence intervals (CI).

Candidate predictors	Description	Early and late dumping symptoms
		OR (95% CI)
Age at surgery	Continuous	0.95 (0.91–0.99)
Biological sex	Male	Reference
	Female	1.55 (0.69–3.49)
Preoperative BMI	Continuous	1.06 (0.98–1.14)
Neoadjuvant therapy	Yes	Reference
	No	3.56 (1.62–7.84)
Charlson Comorbidity Index score	0	Reference
	1	2.26 (0.98–5.19)
Charlson Comorbidity Index score	0	Reference
	≥ 2	1.44 (0.52–3.97)
Surgical approach	Open oesophagectomy	Reference
	Minimally invasive/hybrid	0.41 (0.20–0.84)
Anastomosis location	Thoracic	Reference
	Cervical	2.10 (0.83–5.30)
Postoperative complications	CD IIIb–IV	Reference
	CD 0–I	2.75 (1.02–7.36)
Postoperative complications	CD IIIb–IV	Reference
	CD II–IIIa	1.71 (0.63–4.59)

### The model performance

**Figure 1:**
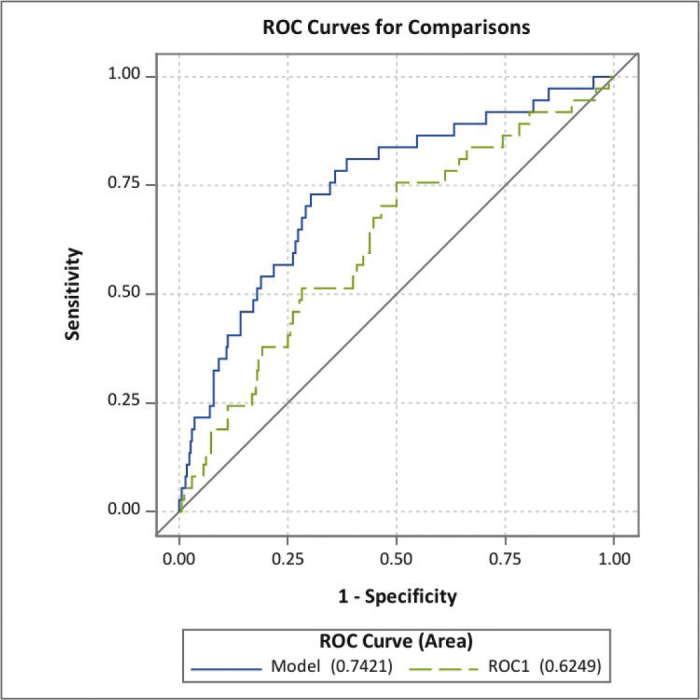
Performance of the prediction model for early dumping symptoms including: younger age, female sex, higher preoperative body mass index, no neoadjuvant therapy, Charlson Comorbidity Index score > 0, no minimally invasive or hybrid surgery, cervical anastomosis, and postoperative complications (lower Clavien Dindo grade) presented as the area under the receiving characteristics curve (AUC) for the full model was 0.74, 95% CI: 0.64–0.83 and cross-validated (ROC1) model (AUC 0.62, 95% CI: 0.53–0.72).

**Figure 2:**
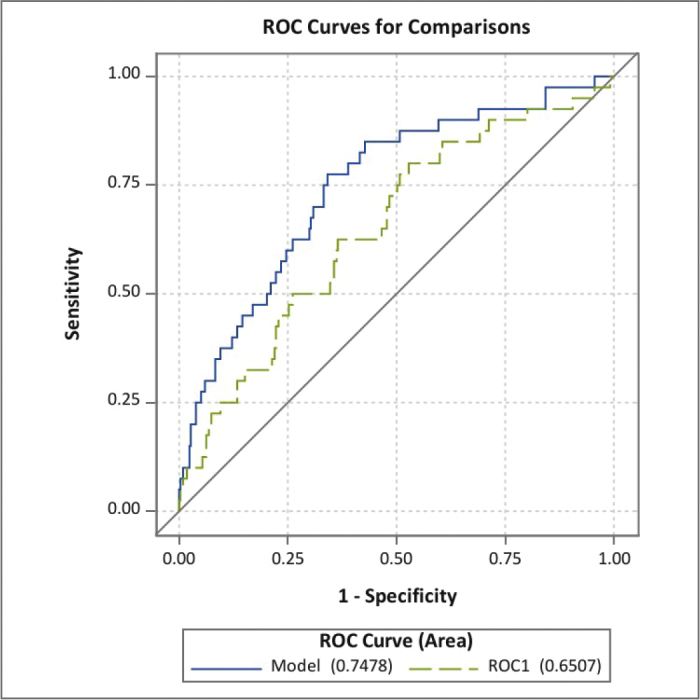
Performance of the prediction model for early and late dumping symptoms including: younger age, female sex, higher preoperative body mass index, no neoadjuvant therapy, Charlson Comorbidity Index score > 0, no minimally invasive or hybrid surgery, cervical anastomosis, and postoperative complications (lower Clavien Dindo grade), presented as the area under the receiving characteristics curve (AUC) for the full model (AUC 0.75, 95% CI: 0.67–0.83) and cross-validated (ROC1) model (AUC 0.65, 95% CI: 0.56–0.74).

For the prediction of early dumping symptoms, the AUC for the total cohort was 0.74 (95% CI: 0.64–0.83), and after cross-validation, the AUC was 0.62 (95% CI: 0.53–0.72) (Figure 1). For early and late symptoms, the AUC was 0.75 (95% CI: 0.67–0.83), and for the cross-validated model, the AUC was 0.65 (95% CI: 0.56–0.74) (Figure 2). The *p*-value for the Hosmer and Lemeshow test was 0.14.

### The CART model

The CART model had 14 terminal nodes (Supplementary Figure 1). The seven most important candidate predictors used in the splits were age at surgery, preoperative BMI, neoadjuvant therapy, Charlson Comorbidity Index score, biological sex, surgical approach, and tumour stage (Supplementary Table 2). The classification accuracy was 90% for training and validation, respectively. The mean square error was 0.07 (training) and 0.11 (cross-validation).

## Discussion

We developed a prediction model to estimate the risk of dumping syndrome 1 year after oesophageal cancer surgery, using routinely collected demographic and clinical variables. The final model demonstrated moderate discriminative performance, with an AUC of 0.74 and an internally validated AUC of 0.62.

This nationwide, population-based cohort study, with a relatively high participation rate (66%), provided a broadly representative sample of surgically treated oesophageal cancer survivors by including all eligible patients in Sweden during a defined time period. Nonetheless, the possibility of selection bias being introduced through differential loss to follow-up cannot be excluded. Information on candidate predictors was obtained through a comprehensive review of prospectively collected data in medical records, and the dumping syndrome assessment was based on well-validated questionnaires. However, no glucose measurements or oral glucose tolerance tests were performed, which limits the ability to verify these symptom-based diagnoses [2, 5]. In addition, there is no universally accepted definition of dumping syndrome following oesophagectomy. Although the accuracy of questionnaire responses was supported by follow-up interviews conducted by a trained research nurse, the subjective nature of symptom reporting also remains a limitation. The lower prevalence of dumping in our study compared with Lin et al. [8] may be explained by our 1-year postoperative assessment, as symptoms often decline over time with adaptation, potentially leading to the risk of underestimation of true prevalence. Differences in nutritional management across studies may further account for variability in the reported prevalence of dumping in the literature and limit direct comparability. In addition, the relatively small number of patients with dumping syndrome in this study (*n* = 41) reduces statistical power and increases the risk of overfitting, despite internal validation. These measurements and sample-size limitations may have influenced model performance, and the findings should therefore be interpreted with caution. External validation in larger cohorts will be important to confirm generalisability.

Few prediction models have focused on identifying factors associated with long-term morbidity following oesophageal cancer surgery, although persistent symptoms often impact survivors’ quality of life [27, 28]. This underscores the importance of developing tools that can support early identification and management of postoperative complications such as dumping syndrome. Our model provides individual risk estimates with modest accuracy using variables available at hospital discharge and may help to distinguish between high- and low-risk individuals. While most predictors included are non-modifiable, this information can guide preventive strategies and tailored follow-up. Currently, there seems to be no available strong predictors of dumping syndrome, although dietary factors appear to play a significant role in symptom manifestation. In this study, a lower Clavien Dindo grade was associated with a higher prevalence of dumping symptoms. This may reflect that patients with less complicated recoveries had resumed normal eating patterns to a larger degree than patients experiencing more severe postoperative complications at 1 year after surgery. Patients with less complicated recoveries may therefore more frequently have been exposed to dietary triggers known to provoke dumping, whereas patients with severe postoperative complications may have experienced delayed oral intake, prolonged dietary restrictions, or more pressing medical issues, overshadowing symptoms of dumping.

Our objective was to identify patients at risk of developing dumping syndrome in the early postoperative period, enabling timely and appropriate clinical interventions. This is particularly important since most patients have not yet resumed normal eating patterns within 30 days following surgery, and nutritional recovery is typically a stepwise process [29]. Therefore, all patients should receive dietary advice after undergoing oesophagectomy. To further improve the predictive accuracy of our model, future research should explore dietary factors as candidate predictors.

To conclude, this nationwide population-based cohort study offers insights into factors associated with dumping syndrome after oesophagectomy. Given the prediction model’s modest ability to distinguish between high‑ and low‑risk patients, it should be regarded as exploratory and hypothesis-generating rather than as a tool for clinical decision‑making. Its potential value lies in complementing clinical judgement, informing structured follow-up, and guiding future research.

## Supplementary Material



## Data Availability

The data that support the findings of this study are available upon request by the corresponding author (AS).
