# Supplementary material for: Prediction of dumping after oesophageal cancer surgery
Source: Acta Oncol. 2026 Mar 5;65:45044. doi: 10.2340/1651-226X.2026.45044 (PMC12973169; doi:10.2340/1651-226X.2026.45044)
Supplement: Supplementary file 1 [file AO-65-45044-s1.pdf]

Supplementary material has been published as submitted. It has not been copyedited, or typeset by Acta Oncologica

Supplementary Table 1. Univariate p-values

**Candidate predictors**	**Model 1**<br><br>**Early dumping symptoms**	**Model 2**<br><br>**Early and late dumpting symptoms**
	P-value	P-value
**Age at surgery**	0.003	0.001
**Biological sex**	0.05	0.06
**Preoperative Body Mass Index**	0.02	0.01
**Neoadjuvant therapy**	0.02	0.02
**Charlson Comorbidity Index score**	0.16	0.15
**Surgical approach**	0.02	0.02
**Anastomosis location**	0.26	0.27
**Postoperative complications**	0.3	0.2

Supplementary Table 2. The variable importance for the candidate predictors

**Candidate predictors**	**Training**	
	**Relative**	**Importance**
**Age at surgery**	1.00	1.85
**Preoperative BMI**	0.83	1.53
**Neoadjuvant therapy**	0.64	1.19
**Charlson Comorbidity Index score**	0.61	1.12
**Biological sex**	0.55	1.02
**Surgical approach**	0.50	0.92
**Tumour stage**	0.36	0.66

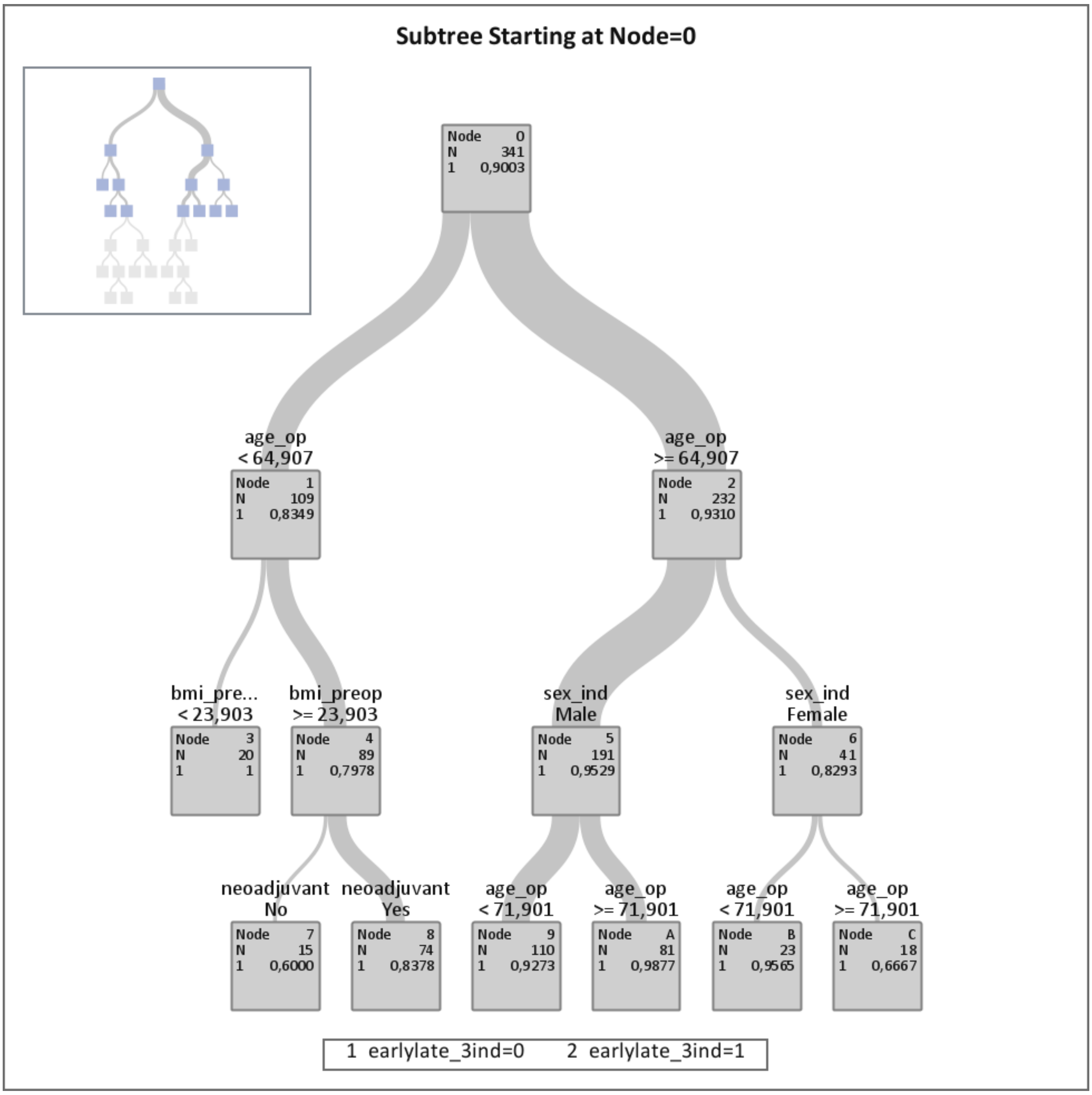

Figure. The CART model of early and late dumping symptoms 1 year after oesophageal cancer resection.
